# Recent Emerging Immunological Treatments for Primary Brain Tumors: Focus on Chemokine-Targeting Immunotherapies

**DOI:** 10.3390/cells12060841

**Published:** 2023-03-08

**Authors:** Alessio Ardizzone, Rossella Basilotta, Alessia Filippone, Lelio Crupi, Marika Lanza, Sofia Paola Lombardo, Cristina Colarossi, Dorotea Sciacca, Salvatore Cuzzocrea, Emanuela Esposito, Michela Campolo

**Affiliations:** 1 Department of Chemical, Biological, Pharmaceutical and Environmental Sciences, University of Messina, Viale Ferdinando Stagno d’Alcontres 31, 98166 Messina, Italy; aleardizzone@unime.it (A.A.); rossella.basilotta@unime.it (R.B.); alessia.filippone@unime.it (A.F.); lelio.crupi@studenti.unime.it (L.C.); mlanza@unime.it (M.L.); salvator@unime.it (S.C.); campolom@unime.it (M.C.); 2 Istituto Oncologico del Mediterraneo, Via Penninazzo 7, 95029 Viagrande, Italy; sofia.lombardo@grupposamed.com (S.P.L.); cristina.colarossi@grupposamed.com (C.C.); dorotea.sciacca@grupposamed.com (D.S.)

**Keywords:** primary brain tumors, immunotherapy, chemokines, chemokines-receptors, chemokine-targeting immunotherapies

## Abstract

Primary brain tumors are a leading cause of death worldwide and are characterized by extraordinary heterogeneity and high invasiveness. Current drug and radiotherapy therapies combined with surgical approaches tend to increase the five-year survival of affected patients, however, the overall mortality rate remains high, thus constituting a clinical challenge for which the discovery of new therapeutic strategies is needed. In this field, novel immunotherapy approaches, aimed at overcoming the complex immunosuppressive microenvironment, could represent a new method of treatment for central nervous system (CNS) tumors. Chemokines especially are a well-defined group of proteins that were so named due to their chemotactic properties of binding their receptors. Chemokines regulate the recruitment and/or tissue retention of immune cells as well as the mobilization of tumor cells that have undergone epithelial–mesenchymal transition, promoting tumor growth. On this basis, this review focuses on the function and involvement of chemokines and their receptors in primary brain tumors, specifically examining chemokine-targeting immunotherapies as one of the most promising strategies in neuro-oncology.

## 1. Introduction

Malignant brain tumors represent 32% of all primary tumors of the central nervous system (CNS), and the remaining 68% are benign neoplasms originating in the CNS (e.g., meningiomas, nerve sheath tumors, and pituitary tumors) [1].

By far, high-grade gliomas are the most common malignant primary brain tumors in adults and include a variety of tumor subtypes in which glioblastomas are the most aggressive form [2].

Overall, the prognosis of malignant primary brain tumors is poor. In fact, after diagnosis, the five-year relative survival rate of primary brain tumors is less than 36%, reaching 6.8% in the case of glioblastoma which is the most common malignant subtype [1].

Currently, the therapeutic approach to malignant primary brain tumors is based on a multimodal approach involving a maximal safe surgical resection as a first step, followed by radiotherapy and conventional chemotherapy with alkylating agents, such as temozolomide (TMZ) [3].

Recent advances in the comprehension of the biology of these tumors have prompted pharmacological research toward the development of new therapeutic strategies, mainly new immunotherapeutic agents. In particular, agents targeting the programmed cell death protein 1 (PD1)—PD1 ligand 1 (PDL1) axis or the cytotoxic T lymphocyte-associated antigen 4 (CTLA4) as well as therapeutic vaccines and chimeric antigen receptor (CAR) T-cell therapy which have been tested at both preclinical and clinical levels [4].

Despite the promising results obtained by pre-clinical studies, most of the innovative treatments failed to show relevant benefit in Phase II/III clinical trials, likely because of the high degree of intratumor heterogeneity of malignant primary brain tumors, and especially the inherent limitations of intracranial drug delivery for glioblastomas [5].

In addition to the above strategies, the chemokine system has progressively gained interest as a potential target for immunotherapy in a variety of cancers, including primary brain tumors [6].

Chemokines are a heterogenous family of low molecular weight secretion proteins that interact with specific membrane chemokine receptors and induce a variety of migratory responses in their target cells, particularly leukocytes. Beyond this role, chemokines play a central role in a variety of physiological processes, such as in the maintenance of immune system homeostasis, immune surveillance, inflammatory responses, and tissue repair [7]. In addition, chemokine receptors can also be expressed by different non-leukocytic cells, and together with their respective chemokines are involved in the onset and/or progression of inflammatory pathologies [8], autoimmune disorders [9], metabolic and endocrine syndromes [10], and cancer [11].

Concerning the latter, there is evidence that some chemokines are specifically involved in the activation/maintenance of pathways related to tumoral hallmarks, mainly by promoting the development of a pro-inflammatory tumor environment.

In addition, it has been shown that chemokines play a role in favoring tumor neo-angiogenesis and chemokine-mediated leukocyte chemoattraction mechanisms and that may explain the occurrence of resistance in subsets of patients treated with angiogenesis inhibitors [12]. Furthermore, chemokines produced by tumor cells or by tumor-associated immune cells may promote cancer progression by modulating apoptosis and/or activating proliferative pathways [6]. From all the above, it is not surprising that the chemokine system has emerged as a valuable target for the development of new therapeutic approaches and that an ever-growing number of agents directed at this system have been recently developed. Therefore, this review aimed to evaluate the recent advances in pharmacological research, especially focusing on the identification and use of new therapeutic agents selectively targeting chemokines and chemokine receptors and their actual therapeutic efficacy in the treatment of malignant primary brain tumors.

## 2. Primary Brain Tumors Hallmarks and Canonical Therapies

### 2.1. Brain Tumors: Classification

Primary brain tumors represent a group of neoplasms that originate from the different cell types of the CNS, which is the most complex and highly organized body system [13].

Brain tumors fall into two large groups: primary and metastatic. Primaries arise from the brain or its membranes, while metastatic, or secondary, originate in other parts of the body and spread to the brain, usually through the bloodstream [14]. Primary tumors can be benign or malignant, while metastatic tumors are always malignant [14].

Benign tumors, such as meningiomas, are usually slow-growing and very well-defined by the surrounding tissues; they can be completely removed by surgery [15]. However, although this type of tumor does not metastasize, this abnormal growth can exert pressure on the sensitive tissues of the brain, altering its functions [16].

Usually, benign tumors are not life-threatening, although the need for surgery still poses a risk of nerve injury for the patient [17]. Conversely, malignant tumors are fast-growing and have the ability to infiltrate surrounding tissues.

CNS cancers include a variety of pathological subtypes, and despite the different classifications proposed over the years, the most appropriate subdivision is based on their origin. Indeed, an accredited distinction divides “non-glial tumors” from “glial tumors” [18].

The first one is typically localized in the posterior cranial fossa and, in adults, they could develop in both hemispheres of the cerebellum, while in the pediatric age group, they are often found in the cerebellar worm [19].

Glial tumors are tumors that originate from glial cells and represent the most common form of all CNS tumors, with an incidence greater than 80% [20].

The current WHO CNS 2021 5th edition (WHO CNS5) is based on the use of complex histological and molecular approaches to establish a final pathological diagnosis and classification for brain tumors [21].

**Figure 1:**
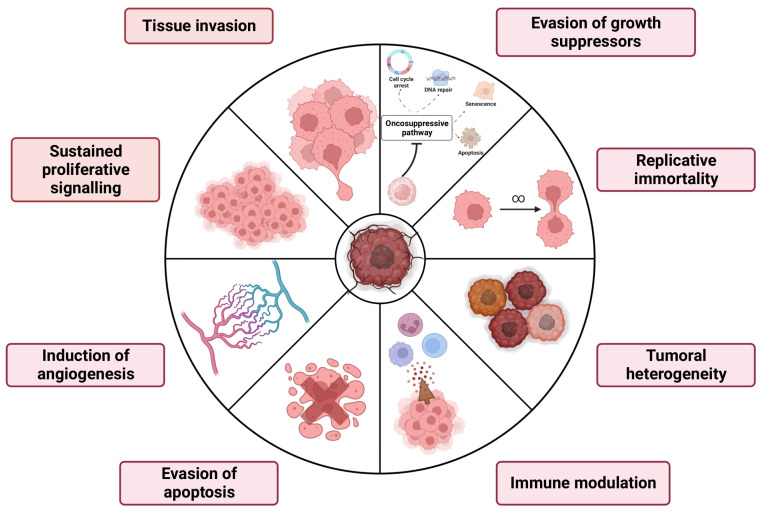
Biological features of primary brain tumors.

Currently “glial, glioneuronal and neuronal tumors” are grouped into a separate family and divided into six categories: (1) diffuse adult-type gliomas, (2) diffuse low-grade pediatric gliomas, (3) diffuse pediatric-type gliomas with high-grade gliomas, (4) circumscribed astrocytic gliomas, (5) glioneuronal and neuronal tumors, and (6) ependymal tumors [21]. Biological mechanisms of brain tumors are summarized in Figure 1.

### 2.2. Brain Tumors: Current Clinical Approaches

Overall, in recent decades, there has been a considerable increase in the incidence of primary brain tumors, mainly affecting those over 65 years of age [22].

In particular, CNS cancers are more present in men than in women, even if some histological types such as meningiomas and pituitary tumors are more frequent in women while gliomas are prevalent in men [23].

The clinical manifestations of this cancer mainly depend on its location and size, causing compression, increased intracranial pressure, and cerebral edema [24]. The current drug therapy is prescribed according to the age of the patient and the site of the neoplasm.

The treatment can be trimodal, defined as a therapeutic approach in which three different treatment strategies converge: radiotherapy, chemotherapy, and surgery [25]. Each of these therapeutic options provides its fundamental contribution to a positive prognosis. In fact, in the treatment of neoplastic pathology, the trimodal therapeutic approach considerably increases the possibility of healing and reduces the risk of relapses [26].

Chemotherapy is successful against some types of cancer, however, in this case, this therapeutic approach is hindered by the presence of the blood-brain barrier (BBB) [27]. The BBB acts as a functional structure interposed between the blood and the nervous parenchyma which selectively regulates the blood passage of chemicals or drugs to and from the brain, protecting the nervous system [28].

Temozolomide, as well as Lomustine and Carmustine, are among the few chemotherapeutics able to cross the BBB [29]. In this regard, recent preclinical studies have shown how innovative distribution systems are promising for the “transport” of therapies across biological barriers, reducing side effects and intervening more specifically with cancer cells [30]. Chemotherapy drug-laden nanocarriers have been shown to be hopeful against glioma tumors [31]. More specifically, a triple conjugated system developed with carbon dots, transferrin, and two anti-cancer drugs (Epirubicin and Temozolomide) was successful in decreasing U87 cell line viability [32].

Likewise, effective results were obtained from the synergistic action of drug therapy with radiotherapy. In fact, the prognosis of patients with low-grade gliomas was considerably improved by combining the three drugs Lomustine, Procarbazine, and Vincristine with radiotherapy [33,34].

These pieces of evidence elucidate the critical role of radiotherapy in the management of several primary brain tumors. In particular, the recent progress in radiation techniques comprises intensity-modulated radiotherapy (IMRT), volumetric-modulated arc therapy (VMAT), and stereotactic radiosurgery (SRS) [35]. These innovative techniques allow for the delivery of higher radiation doses to the target volume while reducing the toxicity to healthy tissues when compared with conventional 3D-conformal radiotherapy (3D-CRT) [35]. Thanks to these advances, the primary care standard for brain cancer patients has notably improved. Recently, immunotherapies have represented a remarkable evolution in the branch of primary brain tumors [36]. For instance, clinical trials validated the effectiveness of antibody-based immunotherapies which appear to be very successful and extremely selective for the target of action, reducing the development of severe side effects, although they represent an important cost for the health system [37].

These studies suggest that the immune system plays a central role in the cancer environment; therefore, it is possible to generate well-timed positive immunomodulatory effects by regulating the production of immunostimulatory cytokines and chemokines.

## 3. Immunotherapeutic Approach to Control Primary Brain Cancers

Immunotherapy is defined as the therapeutic use of methods capable of modulating the functions of the immune system, acting on the cellular and humoral mechanisms that regulate the physiological response to the antigen [38]. The immune system plays an essential role in immune surveillance, as cells of the adaptive and innate immune systems infiltrate the tumor microenvironment (TME), interact with cancer and stromal cells, and contribute to the modulation of tumor progression. Therefore, to date, cancer immunotherapies have been reconsidered and recognized as the fourth method of treatment, after surgery, chemotherapy, and radiotherapy [39]. The goal of immunotherapy is to enhance the natural defenses to eliminate malignant cells, and this has represented a revolution in the field of oncology and cancer treatment [40]. Cancer immunotherapy aims to balance the immune system to eliminate cancer cells without producing uncontrolled autoimmune inflammatory responses that could lead to therapeutic limitations [41]. Immunotherapies are generally classified into active and passive immunotherapies. Active immunotherapy means the direct stimulation of an immune response, immune memory, and a lasting response as in the case of oncolytic vaccines. Such active immunization can generate a non-specific response or a specific response. The non-specific response occurs when the goal is to generate an adaptive host response against malignant cells. The specific response occurs when an antigen (or more precisely an immunogen) is administered in the expectation of generating an antibody, a cytotoxic T lymphocyte (CTL), or a combined response against an antigen associated with a defined tumor. On the other hand, passive immunotherapy involves the administration of an antibody against one or more defined antigens or a reactive lymphocyte that recognizes the malignant tumor cell. This mechanism can produce both non-specific responses, when the goal is to activate an adaptive response of the host, or specific responses, when the target is a targeted response against a particular malignant cell. However, these responses are short-lived and often require regular administration of treatment, as is the case of monoclonal antibodies [42]. Although immunotherapeutic approaches have proven highly effective in various types of tumors, to date, their use in the case of patients with primary brain tumors has shown poor results in terms of survival rate [43]. This is due to the uniqueness of the cell types that distinguish brain tissue and also to the action of the BBB, which contributes to making the brain a relatively immune-privileged organ. Indeed, immune-privileged organs are characterized by tightly regulated immune responses, which consequently cause a naturally immunosuppressive environment. Furthermore, some intrinsic characteristics of primary brain tumors, including the high degree of heterogeneity, also contribute to poor immunogenicity and immunosuppression [44].

### 3.1. Clinical Implication of Vaccines in Brain Cancers

Cancer vaccines use tumor cell lysates, dendritic cells (DC), nucleic acids (such as mRNA), or tumor-specific antigens to trigger anticancer immune responses.

In the design of therapeutic cancer vaccines, the choice of target antigen is of the most importance. Some vaccines use tumor-associated antigens (TAAs), autoantigens that are abnormally expressed by tumor cells but are subject to central and peripheral tolerance mechanisms, which is why the high-affinity T-cell bank for TAAs may be insufficient to elicit an immune response. These are recognized by a high affinity for T lymphocytes and are therefore less susceptible to central tolerance and autoimmunity phenomena. When an artificially synthesized antigenic protein/peptide is administered, it is absorbed by professional antigen-presenting cell (APCs) and presented in a complex with human leukocyte antigens (HLA) molecules on the cell surface, while, when T-cells recognize antigens, cancer-specific immune responses are induced. Since many of the early clinical trials of protein/peptide vaccines reported favorable results, Phase III studies were conducted to confirm the results. Unfortunately, most of these studies have failed, suggesting that single protein/peptide vaccines do not exert sufficient anticancer effects. These unexpected findings can be explained by several factors, including tumor immune escape mechanisms and immunosuppressive TMEs [45].

The development of a brain tumor vaccine faces several challenges arising from rapid growth, tumor heterogeneity, low tumor mutational burden, and immunosuppression due to the activation of brain-resident microglia and macrophages [46]. Tumor-associated macrophages constitute 30% of the tumor mass in glioblastoma, favoring the transition to the M2 immunosuppressive phenotype which inhibits cluster of differentiation (CD) 41 and CD81 T-cell functions and induces regulatory T-cell differentiation. Therefore, this immunosuppressive environment represents the main limitation that determines a poor response to immunostimulant therapies such as therapeutic cancer vaccines. Several peptide vaccines have been developed for the treatment of primary brain tumors. Among these, there is the epithelial growth factor receptor variant III (EGFRvIII) peptide vaccine which targets EGFRvIII, a tumor-specific antigen, highly expressed in glioblastoma, and stimulates the patient’s immune system against EGFRvIII-positive glioblastoma cells; however, it is less successful for EGFRvIII-negative or low-expressing tumor cells. Thus, in a heterogeneous tumor such as glioblastoma, single antigen therapy may have a very limited success rate despite a measurable positive immune response [47].

Some preclinical results demonstrated that peptide vaccines targeting the isocitrate dehydrogenase 1 (IDH1) R132H mutations presented on major histocompatibility complex (MHC) II induced mutation-specific CD41 T-cell activation and antibody production [48]. Another target is the K27M mutations in the histone-3 gene (H3K27M), highly expressed in aggressive midline gliomas. Again, preclinical studies demonstrated that H3K27M peptide vaccines presented via MHC I elicited CD41- and CD81-specific immune responses [49]. Autologous DC vaccines are produced from DCs extracted from patients via leukapheresis and exposed ex vivo to the tumor-associated antigens (peptides or messenger RNA) of choice. The engineered cells are subsequently delivered peripherally or intracranially to the patient to generate a cell-mediated and humoral immune response against various targets, stimulating T-cells to cross the blood-brain barrier. DC-based vaccines have shown significant clinical results due to their ability to present antigens and thereby directly activate T-cells to attack tumor cells. A representative example is sipuleucel-T, a DC-based immunotherapy that has been approved for the treatment of advanced prostate cancer [50]. Furthermore, they have a low toxicity profile and their ability to target multiple antigens simultaneously offers an advantage over single-antigen therapies when it comes to heterogeneous tumors such as primary brain tumors. A multi-peptide DC-vaccine (ICT-107) was tested in a Phase I study, in newly diagnosed glioma patients with HLA-A1 or HLA-A2 and at least 1 TAA such as human epidermal growth factor receptor 2 (HER2), tyrosinase-related protein-2 (TRP-2), glycoprotein 100 (gp100), Melanoma Antigen-1 (MAGE-1), interleukin-13 receptor subunit alpha-2 (IL13-Ra2) or absent in melanoma 2 (AIM-2) in combination with standard chemotherapy and radiation. Overall, this study showed promise, with an increase in median progression-free survival of 16.9 months and median overall survival of 38.4 months [51]. Based on this evidence, another pulsed autologous DC vaccine with known TAAs: IL13-Ra2, EphrinA2, and Survivin (trade name: SL701) was developed. Currently, SL701 with or without Avastin (bevacizumab) is under Phase II investigation (NCT02078648) [52].

### 3.2. Therapeutic Practice of Monoclonal Antibodies in Brain Cancers

Monoclonal antibodies (mAbs) are the most commonly used and approved method of cancer immunotherapy in clinical practice for several types of cancer, including lymphoma, breast, and colon cancer [53]. The antitumor immunotherapeutic efficacy of mAbs is based on three main mechanisms. These mechanisms include (i) the inhibition of factors that activate signaling pathways used in proliferating tumor cells and angiogenesis by antibody binding; (ii) antibody-dependent cellular cytotoxicity (ADCC) composed of target monoclonal antibodies formed from chimeric or fully human antibody components that bind to specific tumor-associated antigens, and (iii) complement-dependent cytotoxicity (CDC) by complement activation [54].

Although mAbs have different mechanisms of action, all of these mAbs have become part of the standard treatment protocol in combination with conventional chemotherapy and/or radiotherapy to overcome some side effects, toxicity, resistance, local variations in blood flow, or ineffective delivery at the target.

The first FDA-approved mAb for cancer immunotherapy, Rituximab, is a chimeric mAb and targets the CD20 antigen in the treatment of B-cell non-Hodgkin lymphomas [55]. The application of mAbs in primary brain tumors is severely limited due to the restriction of the BBB, which makes it extremely difficult to deliver mAb therapies in the CNS. Indeed, the concentration of mAb that can be delivered to the brain is 1000 times lower than that in the bloodstream. Considerable efforts have been made to improve the brain delivery efficiency of mAbs, including fusing mAbs with a peptide or antibody targeting a specific BBB receptor, to improve receptor-mediated transcytosis, or incorporating mAbs within colloidal nanocarriers, for example, nanoparticles or liposomes [56].

Nimotuzumab (Nimo) is a mAb that targets the epidermal growth factor receptor (EGFR) and is in late-stage clinical trials for high-grade glioma. These studies also evaluated the ability of mAb to cross the BBB, showing positive uptake at the known site of tumors. Although the BBB can disrupt the delivery of agents to brain malignancies, surgery, radiation, and the tumor itself disrupt its integrity, allowing the drug to be taken up by tumors. Additionally, in rapidly growing glioma, newly formed blood vessels may lack the BBB function. As a result, nimotuzumab could easily enter the tumor through newly formed intact blood vessels [57].

### 3.3. Generation of Adoptive Cell Therapies to Counteract Brain Cancers

Adoptive cell therapy (ACT) uses genetic engineering to give T-cells the ability to recognize and kill cancer cells, boosting intrinsic immune capacity. Unlike vaccines and immunomodulatory agents which rely on the activation of reactive endogenous tumor cells, ACT offers the ability to genetically select and engineer cells with specificity for tumor antigens in order to achieve therapeutic targets through the appropriate stimulation of potent function effectors. CAR T-cell therapies are based on the extracellular domain resembling an antibody recognizing the TAA of choice fused to intracellular T-cell signaling components [58]. To date, the FDA has approved some therapies with CAR-T-cells, as they have been able to induce remission in several hematologic cancers [59]. T-cells can also be engineered to express a transgenic T-cell receptor (TCR), which plays an important role in the immune response by promoting the killing of infected or foreign cells by cytotoxic T-cells or by promoting the inhibition of the cell response of regulatory T-cells. Clinically, TCR-T-cells have been less extensively studied than CAR-T-cells. Whereas CAR-T-cells are redirected to surface antigens via an antibody-based targeting fraction, TCR-T-cells express a heterodimeric receptor that recognizes antigen-derived peptides presented in the HLA context [60]. TCR cell therapy was first used for the treatment of metastatic melanoma, in which lymphocytes engineered to express TCR capable of recognizing melanocyte differentiation antigen (MART-1) demonstrated beneficial effects in the treatment of the disease [61].

Most studies with CAR-T-cells in GBM target EGFRvIII, IL13Ra2, or HER2. A Phase 1 study demonstrated the safety of a single intravenous infusion of EGFRvIII CAR T-cells, as it did not show off-target toxicity and cytokine release syndrome [62]. In the study by Brown et al., an intracranial injection of IL13Ra2 CAR T-cells was shown to be safe in patients who received the treatment, resulting in tumor regression [63].

Regardless of the antigen recognition modality, T-cell therapy in primary brain tumor patients aims to overcome several limitations. The main challenges are the profound antigen heterogeneity and immunosuppression as well as limitations on lymphocyte homing resulting from the blood-brain barrier and the high degree of relapses and toxicity, as exemplified by the frequent occurrence of cytokine release or neurotoxicity.

### 3.4. Immune Checkpoint Inhibitors

Immune checkpoint inhibitors (ICIs) work by releasing the inhibitory mechanisms that control T-cells, triggering the activation of antitumor immune responses. In addition to invigorating T-cells, ICIs can activate other cells of innate and adaptive immunity, thus obtaining an effective synergistic response against tumors [64]. Immune checkpoints refer to the set of inhibitory pathways that immune cells possess to regulate and control the duration of the immune response by maintaining self-tolerance and limiting autoimmunity. Tumors use checkpoint inhibition to prevent the T-cell-mediated killing of tumor cells; therefore, activation of these checkpoint inhibition pathways activates anergic T-cells and leads to a robust antitumor response [65]. Among the FDA-approved ICIs for use in humans are those targeting three different molecules: CTLA-4 and PD-1 and its ligand PD-L-1. Ipilimumab is a CTLA-4 inhibitor initially approved for the treatment of metastatic melanoma [66] but also extended to other types of cancer [67,68,69]. Otherwise, the PD-1 blockers Pembrolizumab and Nivolumab act through their ligands, PD-L1 and PD-L2, to counteract active T-cell responses [70,71]. Finally, PD-L1 inhibitors have shown greater efficacy and tolerability compared to other classes of ICIs and, among those approved by the FDA, we mention atezolizumab, durvalumab, and avelumab, mainly used for the treatment of urothelial carcinoma [72,73], non-small cell lung cancer (NSCLC) [74,75], and Merkel cell carcinoma [76].

**Figure 2:**
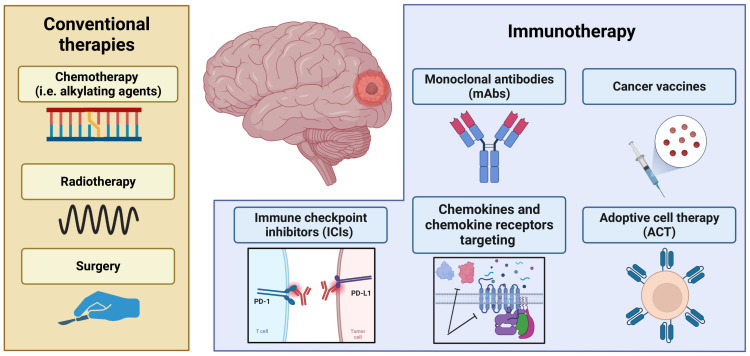
Conventional and innovative therapies to treat brain tumors.

Although antibodies against these molecules are already approved therapies for various types of cancer, several antibodies and small molecules are currently under development that target other immune checkpoints such as Lymphocyte Activation Gene 3 (*LAG3*), T cell immunoreceptor with Ig and ITIM domains (TIGIT), T cell immunoglobulin mucin-3 (TIM3), B7 homolog 3 protein (B7H3), CD39, CD73, adenosine A2A and CD47 [77]. Some checkpoint inhibitors have shown antitumor activity in preclinical glioblastoma studies; however, larger studies have failed to demonstrate a survival benefit. Several experimental antibodies against CTLA-4, indoleamine-pyrrole 2,3-dioxygenase (IDO), and PDL-1 have been tested, both in monotherapy and combination, in mouse models of glioblastoma, showing an improvement in long-term survival associated with an increase in T-cell count and proliferation and a reduction of tumor recurrence in long-term surviving mice, suggesting a role for tumor-directed immune memory [78]. A major study comparing investigational antibodies to CTLA-4, PD-1, PD-L1, and PD-L-2 found that the combination of anti-PD-L1 and CTLA-4 was the most effective, with a 75% tumor-free survival rate in mice [79]. These promising data provided the rationale for subsequent clinical trials testing checkpoint inhibitors in combination with conventional treatments, however, these therapies have thus far failed to demonstrate efficacy in larger Phase III clinical trials. The explanations behind the treatment failure are multiple and may concern impaired interactions between checkpoint inhibitors and PD-1 molecules on the lymphocyte surface, and depletion of functional circulating lymphocytes caused by previous chemotherapy. Furthermore, the BBB can inhibit the release of monoclonal antibodies in the CNS and lymphocytes sequestered within the TME [80]. The novel immunotherapeutic approaches as well as conventional therapies are summarized in Figure 2.

In recent years, advances in the knowledge of tumor immunology have highlighted the centrality of chemokines both in favoring tumor progression and in the development of resistance mechanisms to potentially promising therapies. In this review, we focus on the major chemokines involved in the brain tumor microenvironment, evaluating their expression, regulation, and their role in immune cell recruitment, cancer immunity, and tumorigenesis.

## 4. Chemokines and Chemokine Receptors in Brain Tumors

### 4.1. Chemokines and Chemokine Receptors: Classification and Biological Functions

Chemokines, also known as chemotactic cytokines, are a family of small cytokines having a molecular weight between 8kDa and 12kDa [81]. Chemokines have selective chemoattractant properties to coordinate leukocyte trafficking and their recruitment to sites of inflammation and tissue damage through chemotaxis processes [82].

In recent decades, the key role of chemokines and their receptors has been demonstrated in different biological processes such as embryonic development, hematopoiesis, angiogenesis, and especially the functions of the immune system [83]. Dysregulation of the chemokines’ signaling pathway is involved in the development of many human pathologies, such as autoimmune and chronic inflammatory diseases, immunodeficiencies, and cancer [6,10,84,85].

About 50 chemokines have been identified in humans and are characterized by a protein structure containing cysteines in the N-terminal portion. On the basis of this conformation, they have been classified into four families: CC, CXC, CX3C, and C, where X represents any amino acid and C is cysteine [82].

Chemokines have the characteristic ability to bind different receptors, showing no selective specificity; similarly, receptors can bind multiple chemokines [7]. This property obviously reduces the specificity of pharmacological intervention, aimed at attenuating inflammation which targets chemokines, making them tricky biological targets. Concerning this, the study of chemokines during inflammatory processes can be a useful tool to understand their pathophysiological mechanisms. Chemokines are produced in inflamed tissue by both infiltrating immune cells and tissue-resident cells in response to exogenous and endogenous factors such as lipopolysaccharide (LPS), viruses, autoantigens, and pro-inflammatory cytokines [86,87,88,89]. The latest scientific evidence obtained from in vitro, in vivo, and clinical studies has demonstrated that T helper 1 (Th1) and T helper 2 (Th2) responses are mutually regulated through processes known as re-direction or immune deviation [90,91]. Therefore, interleukin (IL)-12, IL-18, and interferon (IFN)-γ not only favor the development of Th1 cells but inhibit that of Th2 cells [92], while the presence of IL-4 inhibits the development of Th1 cells and causes a shift of the Th1 response towards a less polarized phenotype [93].

The involvement of chemokines in the regulation of Th1/Th2 responses has been widely validated. In fact, chemokines also exert their chemotactic activity towards Th1 and Th2 lymphocytes [94]. Some chemokines seem capable of influencing the polarization of Th1 or Th2 responses by directly interacting with the chemokine receptors present in the cells themselves and/or favoring the production of specific Th1 or Th2 cytokines [95].

Several chemokine receptors associated with immune cells have been described: CCR5 and CXCR3 have been associated with the Th1 phenotype [96], while CCR3, CCR4, and CCR8 have been associated with the Th2 phenotype [97]. The expression of these receptors can change depending on the activated state of T-cells, for example, CCR8 is strongly expressed only by activated Th2 cells.

In this frame, the main stimuli for chemokine production are primary pro-inflammatory cytokines, such as IL-1β, tumor necrosis factor-α (TNF-α), and IL-17 [98]. Furthermore, IFN-γ and IL-4, produced, respectively, by Th1 and Th2 lymphocytes, can induce chemokine upregulation synergistically with IL-1 and TNF-α [99]. These assumptions highlighted inflammation as an essential component of the tumor microenvironment and one of the hallmarks of cancer. Further, it was validated that the crucial role of chemokines as key mediators of cancer-related inflammation is due to their presence at the tumor site for pre-existing chronic inflammatory conditions and acting as triggers of oncogenic pathways [100]. In fact, although chemokines were initially identified as active in determining the composition of tumor stroma, they have been found to directly influence tumor cell proliferation through numerous biological crosstalks [6,101].

Of interest, the CNS is a site of immune privilege but also a complex leukocyte landscape [102]. In particular, tissue-resident populations include microglia and border-associated macrophages (BAMs), which represent the most common phagocytes in the healthy steady-state brain [102]. However, the establishment and progression of a tumoral state stimulates the recruitment of blood-borne monocytes that later differentiate into monocyte-derived macrophages [102]. In this complicated context, the proper recruitment of immune cells is orchestrated by chemokines. In the next sections, the role of each chemokine family in primary brain tumor-related function will be described.

### 4.2. CC Chemokines

The CC or β-chemokines are the largest groups and contain four conserved cysteines.

CC chemokines are an important component of the tumor microenvironment and are produced by tumor cells and tumor-associated cells such as cancer-associated fibroblasts (CAF), tumor-associated macrophages (TAMs), and tumor-associated neutrophils (TANs). CC chemokines increase the proliferation, migration, and invasion of cancer cells while inducing drug resistance. Indeed, inflammatory CC (such as CCL2, CCL3, and CCL5) chemokines join the CCR2^+^ monocytes that differentiate into TAMs at the tumor site [103,104,105]. TAMs possess the dual ability to exert pro- or anti-tumoral activity. CCL20, CCL5, and CXCL12 are effective attractants of dendritic cells (DC) while CCL21 and CCL19 join CCR7^+^ DC in addition to regulatory T-cells (Tregs) [6]. Differently, CCL17 and CCL22 act through CCR4, directly recruiting Tregs and Th2 lymphocytes which both support tumor growth and proliferation [106].

Primary brain tumors such as glioblastoma and glioma are highly vascularized; indeed, the formation of new blood vessels is essential for tumor growth and progression [107]. In the neoangiogenic course, CC chemokines are implicated, favoring tumor growth [12]. Relatedly, it was demonstrated that CCL2, CCL11, CCL16, and CCL18 support tumor angiogenesis as well as endothelial cell survival [108]. Conversely, other chemokines such as CCL21 are able to restrain neoangiogenic processes [109].

Moreover, CC chemokines are involved in many neuropathological processes in which an inflammatory state persists, as well as in brain tumor progression and metastasis. Many chemokines and chemokine receptors are involved in this pathological event. The receptor CCR7 together with ligands CCL19 and CCL21 moderates the migration of tumor cells to lymph nodes [110].

Although the physiological function of CCL14 is still poorly understood, it has been recognized as anti- or pro-cancer in different pathological settings, e.g., the expression of this chemokine is reduced in many solid tumors including liver, breast, lung, and prostate cancer but is elevated in primary brain tumors and esophageal cancer [82]. Likewise, in primary brain tumors, CCL2 participates in the recruitment of neural progenitor cells and microglia [82]. Indeed, in glioblastoma, CCL2, CCL7, and CCL17 were notably increased [111]. In this regard, CCL2 inhibitors or CCR2 antagonists have shown promising results in preclinical models of gliomas [112,113], while other subtypes of CC chemokines are still under investigation.

### 4.3. CXC Chemokines

Similarly to CC chemokines, CXC or α-chemokines are one of the major groups of chemokines. CXC (such as CXCL1, CXCL2, CXCL5, CXCL6, and CXCL8) at the tumor site involve CXCR2^+^ neutrophils that differentiate into TANs [103,104,105]. TANs species have the capacity to exert pro- or anti-tumoral activity. Moreover, the presence of some chemokines at the tumor site can influence leukocyte activation, as in the case of CXCL16, which induces macrophage polarization towards a pro-tumoral phenotype in solid tumors thanks to its binding with CXCR6 [114]. In addition, CXCL9 and CXCL10 are highly linked with the Th1 immune response by recruiting natural killer (NK) cells, with CD4^+^ Th1 and CD8^+^ cytotoxic lymphocytes having the ability to elicit antitumoral responses [115]. In the neoangiogenic course, essential for tumor progression, CXC chemokines are highly involved, favoring tumor expansion [12]. The presence or absence of a glutamic-leucine-arginine (ELR) motif at the N-terminal divides the CXC chemokines into ELR^+^ chemokines with angiogenic activity and ELR^−^ chemokines with angiostatic effects [116]. For instance, CXCL8 favors tumor angiogenesis by stimulating the survival of endothelial cells [108]. Specifically, CXCL16, thanks to its interaction with CXCR6, acts as one of the most potent angiogenic mediators [117]. In a similar way, CXCL12 supports neoangiogenesis while inhibiting endothelial cell apoptosis by binding the receptor CXCR4 present on tumor vessels, or indirectly stimulating leukocyte recruitment [118]. Otherwise, other chemokines comprising CCL21 and ELR^−^ chemokines, such as CXCL4, CXCL9, CXCL10, and CXCL1, can inhibit angiogenic stimuli and endothelial cell proliferation [109]. Neoangiogenesis is strictly correlated to tumor growth and proliferation, which leads to several complications such as interstitial pressure and cerebral edema.

In this neuronal stream, tumors produced a large number of chemokines that, binding their receptors, directly favor cancer cell expansion through diverse signaling pathways such as the phosphatidylinositol-3 kinase/AKT/nuclear factor kappa B (PI3K/AKT/NF-κB) and mitogen-activated protein kinase/extracellular signal-regulated kinase (MAPK/ERK) pathways [119,120]. In addition, chemokine overload at the tumor site can support cells’ survival, modulating apoptotic cascade by the regulation of pro- and anti-apoptotic species; specifically, the downregulation of B-cell lymphoma 2 (Bcl-2) levels or the inhibition of caspase expression [121].

The infiltration of cancer cells in the brain microenvironment constrains the anatomical structures and, over time, triggers and shapes the metastatic process. Overall, the central player of the metastatic processes is the CXCL12/CXCR4 axis. Indeed, the presence of CXCR4 overexpression strengthens the ability of several tumor types to migrate and metastasize into organs while also secreting high levels of CXCL12, a known attractant of cancer cells [122]. CXCL12 activates the CXCR4 receptor, which is expressed in a variety of neural cells, resulting in various biological effects [123]. The CXCL12/CXCR4 path induces the migration and proliferation of cerebellar granule cells and chemoattracts microglia while stimulating cytokine and glutamate release by astrocytes [123]. In addition, the CXCL12/CXCR4 axis was shown to be crucial in grade IV astrocytomas compared to other low-grade tumors [124]. Moreover, it was demonstrated that the CXCL12/CXCR4 pathway is overexpressed in histopathological specimens of human glioblastoma [123].

### 4.4. CX3C Chemokines

The CX3C subfamily, to which CX3CL1 or fractalkine belongs, has three amino acids between the two cysteines and is largely monomeric, but can form dimers similar to chemokine CC12. It is a chemokine involved in the antitumor function of lymphocytes, mainly NK cells, T-cells, and dendritic cells, and also interacts with TAMs, myeloid-derived suppressor cells (MDSCs), and microglia [125]. The CX3CL1-CX3CR1 axis has important functions in the brain. The expression of CX3CR1 occurs in microglia while nerve cells have CX3CL1 expression, which enables microglial chemotaxis to nerve cells [126]. Some studies have shown that in glioblastoma with high expressions of CX3CR1 and CX3CL1, the activation of this axis can lead to the inhibition of tumor cell migration [127].

### 4.5. C Chemokines

The C chemokines family, which comprises chemokine lymphotactin 1 (XCL1) chemokine lymphotactin 2 (XCL2), is structurally distinguished from the other chemokine subfamilies by the presence of only two of the four conserved cysteine residues [128].

Subfamily (X)C, to which XCL1/lymphotactin belongs, lacks the first and third cysteines and structurally forms the canonical chemokine fold in equilibrium with a four-stranded β-sheet that associates into a head dimer tail. C chemokines chemoattract lymphocytes but not neutrophils or monocytes [128]. To date, there are very few studies describing the functions of the XCL1 or its receptor XCR1 in the nervous system. However, the role of XCL1-XCR1 in nociceptive processing has been identified, demonstrating XCR1 upregulation at sites of nerve injury and identifying XCL1 as a modulator of central excitability [129]. Lymphotactin can induce T or NK cell migration in vitro and in vivo and the expression of the gene for this chemokine by tumor cells would appear to have an important influence on the induction of tumor-specific immune responses [130]. For example, NK cell activity is frequently altered in patients with primary brain tumors because of the immunosuppressive factors released by tumor cells. Böttcher et al. described that tumor-secreting inflammatory mediators such as cyclooxygenases (COX) and prostaglandin E2 (PGE2) can suppress NK cell antitumor activity [131]. These interesting data exposed the function of NK cells in enrolling dendritic cells into the tumor microenvironment by releasing the XCL1 chemokine [131].

**Figure 3:**
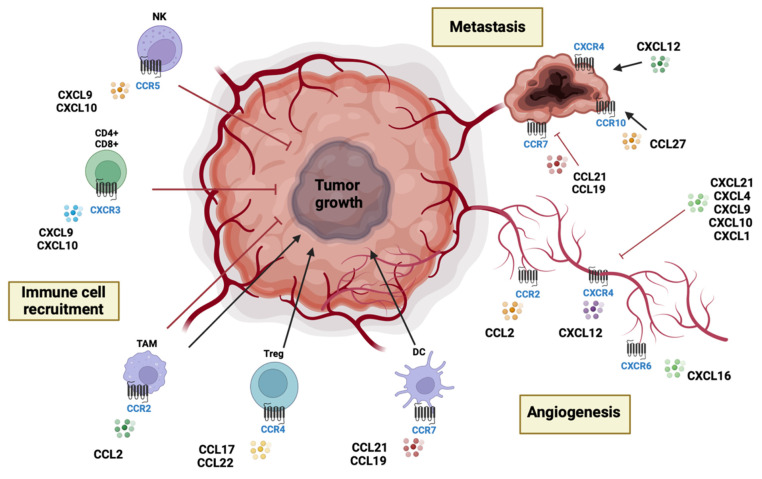
Schematic representation of the role of chemokines and chemokine receptors in brain tumors.

Hence, chemokines and chemokine receptors play important roles in numerous tumor-specific biological processes, as illustrated in Figure 3, not only ensuring the arrival of leukocytes at the sites of inflammation but largely contributing to the non-physiological alterations in various tumor types. These noteworthy biological activities make chemokines attractive targets for pharmacological interventions as well as reliable biomarkers in these pathological conditions.

## 5. Chemokine-Targeting Therapies in Primary Brain Cancers

Over recent decades, emerging findings have elucidated that the chemokine system could represent a promising target for immunotherapy in cancer patients. As previously stated, chemokines have been seen to alter the expression of cancer controlling leukocyte recruitment and activation as well as angiogenesis and cancer cell proliferation in all stages of tumor development. Here, we will discuss the most recent advances in the modulation of the chemokines/chemokine receptors axis, both as a monotherapy or in combination with canonical or immuno-mediated therapies, highlighting its huge potential for the treatment of various brain tumors.

### 5.1. Chemokine-Targeting Therapies: Preclinical Studies

Recent data has proved that glioma tumor stem-like cells support tumor angiogenesis and vasculogenesis via the CXCR4. Therefore, considering the pivotal role of CXCR4 in the context of brain tumors, most research efforts have been aimed at its effective targeting.

A nanocarrier conjugated lipophilic thiobenzoate complex of rhenium-188 loaded in the core of a lipid nanocapsule, containing a CXCR4 function-blocking antibody named 12G5 (12G5-LNC^188^Re), was investigated by Séhédic and colleagues in an in vivo model of glioblastoma [132].

The authors demonstrated the beneficial effect of this nanocarrier by using an orthotopic and xenografic of a U87 cells-induced glioblastoma model [132]. The single infusion of 12G5-LNC^188^Re led to a significant improvement of the outcomes and in particular to the improvement of the median survival, thus suggesting that loaded radiopharmaceutical-nanocarriers could have significant clinical application [132].

Similarly, in their in vitro study, Egorova et al. assessed the effectiveness of modular peptide carriers bearing a CXCR4 targeting ligand [133]. The obtained data demonstrated that peptide carriers modified with CXCR4 ligands are a favorable method to remodel the targeted siRNA delivery systems into CXCR4-expressing cancer and endothelial cells [133]. The CXCR4 antagonist AMD3100 had the capacity to increase the effect of Vatalanib, a vascular endothelial growth factor (VEGF) R2 tyrosine kinase inhibitor, counteracting the growth and cell migration in an in vivo model of glioblastoma [134].

The combination of RS3594, a dual MDM2/4 inhibitor, and AMD3100 has been successful in reducing the invasiveness and migration of glioblastoma cells. In particular, Daniele et al. indicated that the sensitization of glioblastoma cells by AMD3100 increased the antiproliferative activity of RS3594 [135]. In addition to demonstrating the efficient synergistic action of the two compounds, these in vitro data confirmed the blockade of the CXCR4/MDM2/4 axis as a valid strategy to reduce glioblastoma proliferation [135].

Likewise, combined immunotherapy with anti-CXCR4 and anti-PD-1 antibodies conferred a noteworthy survival advantage compared to monotherapy treatment, also modulating tumor-infiltrating leukocytes in the glioblastoma microenvironment as evaluated by an in vivo model [136].

Therefore, since the overactivation of the chemokine receptor CXCR4 is extremely harmful in glioblastoma, drug designers have developed targeted pharmacological agents.

Luo et al. designed and synthesized a novel CXCR4 inhibitor, known as CPZ1344, and examined its antitumor functions [137]. The results obtained in their in vitro study showed how CPZ1344, in a concentration-dependent manner, induced apoptosis mechanisms, thus reducing the growth of glioblastoma cells [137]. Furthermore, CPZ1344 inhibited U87 cell migration and angiogenesis, leading to cell cycle arrest in the G1 phase as well as inhibition of CXCR4 signaling, thereby demonstrating the antitumor effects of CPZ1344 and proposing it as a possible new treatment for glioblastoma patients [137].

Another study evaluated the properties of a new specific CXCR4 antagonist named peptide R [138] by both in vitro and in vivo evaluations. The peptide was obtained by a ligand-based approach and tested through in vitro and in vivo models of glioblastoma [138]. Peptide R impaired the metabolic activity and cell proliferation of the U87 cell line, reducing CXCR4 expression and cell migration in vitro while decreasing tumor cellularity and modulating M1 features in the orthotopic in vivo model [138].

In addition, the novel CXCR4 antagonist, PRX177561, has been proven to regulate the CXCL12/CXCR4 pathway in both in vitro and in vivo models of glioblastoma [139]. PRX177561 decreased cell proliferation, increased apoptosis, and reduced CXCR4 expression and cell migration in response to stromal cell-derived factor 1alpha (SDF-1α) in vitro. In an in vivo model, PRX177561 reduced the weight of subcutaneous tumors while increasing mice’s overall survival [139]. Moreover, PRX177561, co-administered with anti-VEGF/VEGFR therapies such as bevacizumab or sunitinib, exhibited superior antitumor activity, synergistically reducing tumor inflammation and growth by both in vitro and in vivo assessments [140].

Among the several molecular signaling pathways, the expression of SDF-1α/CXCR4 is also a key determinant of other glioma subtypes, causing devasting tumor progression and invasiveness [141].

In this regard, the novel-designed peptide NT21MP of viral macrophage inflammatory protein II (vMIP-II) targeted CXCR4 and inhibited SDF-1α activation in a vitro model of glioma [142]. Particularly, NT21MP, by the inhibition of SDF-1α, prevented cell proliferation, migration, and invasion, upregulated pro-apoptotic factors such as apoptosis-related BCL2 antagonist/killer 1 (Bak1) and Caspase-3, and downregulated cell cycle regulators, arresting the cell cycle in the G0/G1 phase and promoting mechanism of apoptosis [142]. In the orthotopic model, CXCR4 antagonism by POL5551 combined with the anti-VEGF antibody B20-4.1.1 decreased glioma invasiveness and vascular density, resulting in a valuable strategy to overcome antiangiogenic therapy resistance, as revealed by both in vitro and in vivo experiments [143].

Beyond CXCR4, miR-21 has also been extensively identified as a key regulator in glioma malignancy through in vitro and in vivo tests. Relatedly, it was demonstrated that miR-21 or CXCR4 inhibition, but especially their double-targeted knockdown, diminished migration, invasiveness, and proliferation, and enhanced apoptosis in glioma cells by suppressing the PI3K/AKT and Raf/MEK/ERK pathways [144].

The CXCR4 chemokine pathway was also well-validated in other forms of primary brain cancer such as medulloblastoma and neuroblastoma.

Regarding this, Ward et al. indicated the potent antitumor effects due to the dual inhibition of the Sonic hedgehog (SHH) and CXCR4 pathways in a murine model of SHH-subtype medulloblastoma [145].

Additionally, Klein and colleagues provided interesting in vitro and in vivo data describing how CXCR4 signaling controls neuroblastoma tumor growth and response to therapy [146]. BL-8040, a high-affinity CXCR4 antagonist, was able to prevent tumor growth and reduce tumor survival cells. Such effects were traced to the upregulation of miR-15a/16-1, downregulation of their target genes Bcl-2 and cyclin D1, and inhibition of ERK by BL-8040 administration.

Moreover, since the CXCL12/CXCR4 oncogenic bridge serves as communication between tumor cells and stromal cells, numerous molecules targeting CXCL12/CXCR4 signaling have been developed to interfere with tumor growth [147].

Recently, alginate/chitosan nanoparticles entrapping CXCL12 were characterized and probed by in vitro analysis. The system, having an average size of around 250 nm, entrapped CXCL12 with high efficiency (98%) [148]. Thus, the development of an efficient and tunable CXCL12 delivery system could represent a promising therapeutic strategy to be injected into a hydrogel employed to fill a cavity after surgical tumor resection [148]. This system could be used to attract infiltrated glioblastoma cells prior to their removal by traditional treatment, without damaging healthy brain tissue [148].

In a similar manner, a novel SDF-1α inhibitor named olaptesed pegol (OLA-PEG, NOX-A12) reversed the recruitment of macrophages and potentiated the antitumor effect of anti-VEGF antibody therapy such as Bevacizumab or B-20 in an in vivo study [149]. In this orthotopic model, animals treated with OLA-PEG associated with bevacizumab or B-20 manifested a prolonged survival rate compared to anti-VEGF therapy alone [149].

To date, CXCR2 has been linked with numerous biological cell events, comprising inflammation, neovascularization, and cell carcinogenesis [150]. In particular, CXCR2 overexpression was directly related to poor prognosis and recurrence in human glioma [150]. In a preclinical study, CXCR2-expressing cancer cells were found to drive resistance to antiangiogenic therapy in glioblastoma [151]. Indeed, the CXCR2 inhibitor called SB225002 led to reduced tumor growth as well as incomplete vascular mimicry structures in animal models [151]. In the in vivo model created by injecting GL261 glioma cells, CXCR2-blocking by SB225002 notably reduced tumor volume by around 50% [152]. In addition, in the data obtained from their in vitro study, the CXCR2-antagonist at higher concentrations was shown to have a significant impact on endothelial cells [152].

Further investigation on the ability of SB225502 to influence angiogenic factors was performed by Urbantat et al. [153]. In their in vitro study, SB225002 in combination with one of the most used drugs in anticancer therapy, temozolomide, demonstrated a good ability to induce morphological changes in Human Umbilical Vein Endothelial Cells (HUVECs) while downregulating antiapoptotic Bcl-2 expression [153].

These results shed light on the importance of the CXCR2/CXCL2 axis in primary endothelial cells during cancerous events such as glioblastoma, also indicating the need for further investigations in future studies.

In vitro, CCLs such as CCL2 and CCL5 exhibited a meaningful effect on the attraction and migration of glial tumor cells [154,155]. In glioma U251 cells transfected with CCL2 siRNA, there was decreased cell proliferation, cell cycle arrest, and a significant increase in apoptosis-associated proteins such as Caspase-3 and Caspase-7 [156]. In addition, CCL2 inhibition by mNOX-E36 combined with bevacizumab exerted a suppressing effect on the recruitment of tumor-associated macrophages and angiogenic processes in a rat glioblastoma model [112]. Therefore, the inhibition of CCL2 could represent an interesting therapeutic target that would confer a double beneficial effect thanks to the inhibition of the recruitment of CCL2-dependent macrophages as well as modulation of the angiogenic course.

On other hand, CCL5 as an inflammatory mediator also seems to be implicated in the glioma-related developmental process as revealed by the in vitro study of Yu-Ju Wu and colleagues. Jointly with matrix metalloproteinase-2 (MMP2), CCL5 regulates the migratory and invasive flow of glioma cells and consequently increases intracellular calcium levels as well as p-calcium/calmodulin-dependent protein kinase II (p-CaMKII) and p-AKT expressions [155]. However, these harmful effects are alleviated by CCL5 antagonism [155].

Albeit to a lesser extent, some research evaluated the correlation of other chemokines with primary brain tumors, also indicating possible successful targeted therapies.

This is the case of CCR5 blockade by the pharmacological agent maraviroc, which led to a reduction of microglia migration by preventing the M2 microglia phenotype and modulating the AKT pathway in a vitro model [157]. Furthermore, the chimeric anti-ACKR3/CXCR7 antibody, in combination with temozolomide, significantly reduced the tumor mass while prolonging overall survival in a murine model of glioblastoma [158]. Additionally, monotherapy with CCX872, a CCR2 antagonist, improved median survival in an orthotopic model induced by KR158 glioma cells; these antitumor effects were further enhanced by the combination of CCX872 with anti-PD-1 [113].

In addition, very recent discoveries warned about the injurious function of CXCLs in glioblastoma. Looking further, the overexpression of CXCL1 and CXCL2 was closely related to glioblastoma’s aggressiveness, facilitating the migration of myeloid cells and, simultaneously, disrupting the accumulation of CD8^+^ T-cells at the tumor site, thus instigating cancer progression [159].

Likewise, Wang et al. by using in vitro and in vivo assessments, advised CXCL11-armed oncolytic adenovirus as an improvement for immune-virotherapy as well as a promising adjuvant of CAR-T therapy for glioblastoma [160].

All these new compounds represent potential therapeutic approaches against primary brain tumors (as highlighted in Table 1) to be further explored in more complex pre-clinical settings as well as clinical trials.

### 5.2. Chemokine-Targeting Therapies: Clinical Studies

Clinically, Rios and colleagues described a case of a glioblastoma patient treated with AMD3100 (plerixafor) and a combination of a mammalian target of rapamycin (mTOR), a Sirtuin-1 (Sirt1), and an EGFRvIII inhibitor after conventional chemo-radiotherapy [161]. AMD3100 was found to restrain glioblastoma vasculogenesis, specifically blocking the migration of bone marrow-derived cells to the primary tumor site and inhibiting the CXCR4/SDF-1 axis [161].

Moreover, an important overexpression of VEGF was observed in glioblastoma patient samples and was considerably associated with a negative survival outcome [162]. In this regard, it was seen that the proliferation and migration of HUVECs were increased by VEGF, CXCL2, and IL-8 [162].

However, CXCR2 antagonist treatment reduced chemokines and the sprouting of endothelial cells, proving the impact of this pathway in the glioblastoma angiogenic course [162].

Given the excellent clinical results of the patient, AMD3100 could be considered an adjuvant treatment for glioblastoma.

In a Phase I/II clinical study, the intravenous infusion of plerixafor in patients with glioblastoma ameliorated local control of tumor recurrences, constituting a valid adjunct to standard chemo-radiotherapy.

From a clinical perspective, high-grade glioma patients treated with a combination of plerixafor 320 μg/kg and bevacizumab 10 mg/kg well-tolerated the therapy, however, in humans this care option revealed limited efficacy [163].

A clinical trial carried out by Thomas et al. [164] included 29 patients (Phase I = n:9; Phase II = n:20) with glioblastoma who underwent radiation therapy followed by intravenous treatment with the CXCR4 inhibitor plerixafor. The results of the study showed that the treatment was well-tolerated, as it was not associated with severe drug-attributable toxicity, and showed efficacy in decreasing the local tumor recurrence rate. Although the study included a limited number of patients, the treatment proved to be effective in improving the progression free survival (14.5 months) and overall survival (21.3 months) of the patients.

A study by Urbantat et al. [162] explored the role of the chemokines CXCL2 and IL-8 in glioblastoma (GBM) angiogenesis. For this purpose, brain tissue samples from control patients were compared with brain samples from GBM patients. A significant increase in the number of cells expressing CXCL2 and VEGF when compared to controls was observed by immunofluorescence staining. Furthermore, possible correlations between the gene expression of several proangiogenic factors and the overall survival of GBM patients were analyzed using data from the TCGA (The Cancer Genome Atlas) database. In this regard, a significantly worse overall survival was observed in patients with an upregulated expression of CXL2 and IL-8.

Comprehensively, all these data describe the complex and multiple mechanisms through which chemokines and their receptors influence the brain tumor microenvironment (as summarized in Table 1 and Table 2), also highlighting the considerable scientific research progress made over recent decades to find the most targeted and effective therapies.

## 6. Conclusions

Several therapeutic strategies have been developed to modulate the functions of the immune system in order to obtain an advantage in anticancer therapy. Among these are therapeutic vaccines, monoclonal antibodies, immune checkpoint inhibitors, and adaptive therapies. Nonetheless, many of the new immunotherapies fail to achieve the desired results, especially in the clinical phase, due to the numerous challenges that these forms of cancer pose. The most common complications are represented by the poor overcoming of the BBB by these molecules, heterogeneity, and the high mutational burden characteristic of these tumors. The understanding of the role of chemokines and their receptors in the modulation of the biological processes underlying tumor progression has led to their consideration as potential pharmacological targets but has not yet been fully exploited. Thus, the search for new potential therapeutic agents that target chemokines, and their receptors, is a thriving area. To date, as widely discussed, the results obtained by the use of new immunotherapeutic drugs in monotherapy or combined for primary brain tumors appear extremely promising. Therefore, it is likely that more of these therapeutic agents will be developed in the near future, thus constituting valuable support in the fight against primary brain tumors and increasing the survival of cancer patients.

## Figures and Tables

**Table 1:** Summary of the main inhibitors of each molecular target, as reported in every preclinical article.

First Author	Year	Molecular Target	Analysis/Outcome	References
Séhédic D.	2017	CXCR4	The study analyzed the efficacy of rhenium-loaded nanocapsules expressing on their surface an anti-CXCR4, function-blocking antibody (12G5-LNC^188^Re) in an orthotopic in vivo GBM model.	[132]
Shaaban S.	2016	CXCR4	The study evaluated whether whole body irradiation (WBIR) or a CXCR4 antagonist (AMD3100) potentiates the efficacy of vatalanib in an orthotopic in vivo GBM model.	[134]
Daniele S.	2021	CXCR4	The in vitro study evaluated whether CXCR4 inhibition enhances the sensitivity of glioma cells to MDM2/4 inhibitors.	[135]
Wu A.	2019	CXCR4	The study evaluated combination therapy with anti-CXCR4 and anti-PD-1 therapeutic antibodies in an in vivo murine glioma model.	[136]
Luo Z.	2020	CXCR4	The study evaluated the efficacy of a novel CXCR4 inhibitor in a in vitro GBM model.	[137]
Mercurio L.	2016	CXCR4	The study evaluated the effects of a novel CXCR4 antagonist (Peptide R) against glioblastoma in vitro and in vivo.	[138]
Gravina G.L.	2017	CXCR4	The effects of a CXCR4 inhibitor (PRX177561) were evaluated in vitro, on several different glioblastoma cell lines, and in vivo, using a murine xenograft model.	[139]
Gravina G.L.	2017	CXCR4	The study evaluated the efficacy of combination therapy with bevacizumab, sunitinib, and an anti-CXCR4 molecule (PRX177561) by using in vitro models as well as in vivo xenograft murine models.	[140]
Yang Q.	2017	CXCR4	The in vitro study evaluated the effects of a novel designed CXCR4-inhibiting peptide called NT21MP, derived from vMIP-II, on glioma cell lines.	[142]
Liu F.	2020	CXCR4	The study analyzed the effects of the inhibition of miR-21 and/or CXCR4 in vitro on glioma cells and in vivo in murine xenograft glioma models.	[144]
Ward S.A.	2017	CXCR4	The study evaluated the therapeutic potential of dual CXCR4 and SHH inhibition in the treatment of medulloblastoma in an in vivo murine model.	[145]
Klein S.	2017	CXCR4	The study analyzed the role of CXCR4 in neuroblastoma growth and the therapeutic potential of CXCR4 inhibition in neuroblastoma treatment both in vitro and in vivo.	[146]
Gascon S.	2020	CXCR4	The study explored a novel therapeutic strategy for GBM treatment, using non-toxic CXCL12-loaded alginate/chitosan-based nanoparticles. The effects and toxicity of nanoparticles were tested in vitro.	[148]
Deng L.	2017	CXCL12	This study evaluated in an orthotopic in vivo model whether OLA-PEG or NOX-A12 enhanced the antitumor effects of anti–VEGF therapeutic agents.	[149]
Acker G.	2019	CXCR2	The study investigated the role of the CXCR2 pathway in glioma biology and the therapeutic potential of its inhibition using both in vitro and in vivo models.	[152]
Urbantat R.M.	2022	CXCR2	The in vitro study analyzed the proangiogenic pathways following combined treatment with temozolomide and SB225002, a CXCR2 inhibitor, using primary endothelial cells which mimicked the GBM tumor microenvironment.	[153]
Dery L.	2021	CXCL10, CCL2 and CCL11	In vitro and in vivo evaluation of three chemoattractants, CXCL10, CCL2, and CCL11, released by a biodegradable hydrogel (GlioGel) to produce a migration of tumor cells toward a therapeutic trap.	[154]
Yu-Ju Wu C.	2020	CCL5	Using an in vitro model, this study investigated the mechanisms by which CCL5 facilitates the migratory and invasive activity of human glioma cells.	[155]
Lu B.	2017	CCL2	The study evaluated the effects of transfection with a CCL2 siRNA into a human glioma cell line.	[156]
Cho H.R.	2019	CCL2	Using a CCL2 inhibitor in an in vivo murine model, the potential value of CCL2 inhibition in combination with anti-VEGF agents in GBM was studied.	[112]
Laudati E.	2017	CCR5	The authors investigated in vitro the effects of a CCR5 receptor blockade on microglia-glioma interaction through the use of maraviroc, a CCR5 blocker.	[157]
Salazar N.	2018	CXCR7	The study analyzed the safety and efficacy of a single chain FV-human FC-immunoglobulin G1 antibody, X7A, to target ACKR3 in in vivo and in vitro GBM models.	[158]
Flores-Toro J.A.	2020	CCR2	This study evaluated the combination of a PD-1 blockade and CCR2 inhibition in anti-PD-1-resistant gliomas using an in vivo murine model.	[113]
Wang G.	2022	CXCL11	This in vivo and in vitro study investigated the activity of an oncolytic adenovirus (oAds) expressing the chemokine CXCL11 on the infiltration of CAR-T-cells and the reprogramming of the immunosuppressive tumor microenvironment.	[160]

**Table 2:** Summary of the main outcome of every clinical article.

First Author	Year	Molecular Target	Analysis/Outcome	References
Thomas R, P.	2019	CXCR4	Phase I/II study to evaluate the safety and efficacy of “Macrophage Exclusion after Radiation Therapy”, using plerixafor, in glioblastoma patients.	[164]
Lee E, Q.	2018	CXCR4	Phase I clinical study to determine the safety of the combination therapy of bevacizumab and plerixafor in patients with high-grade glioma.	[163]
Rios A.	2016	CXCR4	Case of a patient (66 y.o. male) treated after standard chemo-radiotherapy with combination therapy including a CXCR4 inhibitor, plerixafor, showing a significant clinical response.	[161]
Urbantat R, M.	2021	CXCL2/IL-8	Evaluation of CXCL2 and IL-8 expression in GBM patients’ brain tissue. Analysis of correlations between the gene expression of proangiogenic factors and patients’ overall survival	[162]
